# Key Characteristics and Perception of Different Outbreak Surveillance Systems in Côte d’Ivoire: Cross-Sectional Survey Among Users

**DOI:** 10.2196/56275

**Published:** 2024-07-30

**Authors:** Marta S Palmeirim, Clarisse A Houngbedji, Tanja Barth-Jaeggi, Jean-Pierre Y Kouamé, Aboubakar Krouman, Daouda Coulibaly, Kaspar Wyss

**Affiliations:** 1 Swiss Center for International Health, Swiss Tropical and Public Health Institute, Allschwil, Switzerland; 2 University of Basel, Basel, Switzerland; 3 Centre d’Entomologie Médicale Et Véterinaire (CEMV), Université Alassane Ouattara, Bouaké, Cote D'Ivoire; 4 Centre Suisse de Recherches Scientifiques en Côte d’Ivoire (CSRS), Abidjan, Cote D'Ivoire; 5 National Institute of Public Hygiene, Abidjan, Cote D'Ivoire

**Keywords:** outbreak surveillance system, COVID-19, Côte d’Ivoire, SORMAS, MAGPI, DHIS2, outbreak surveillance, key characteristics, users’ perception, infectious disease, infectious diseases, public health, disease surveillance, policy decision, cross-sectional study, descriptively analysis, Policymakers, health officials, healthcare system, Surveillance Outbreak Response Management and Analysis System, District Health Information Software 2, policy makers, health care system

## Abstract

**Background:**

Accurate and timely infectious disease surveillance is pivotal for effective public health responses. An important component of this is the disease surveillance tools used. Understanding views and experiences of users is crucial for informing policy decisions and ensuring the seamless functioning of surveillance systems.

**Objective:**

In this study, we aimed to assess the user perceptions of 3 disease surveillance tools used in Côte d’Ivoire, namely, MAGPI, District Health Information Software 2 (DHIS2), and Surveillance Outbreak Response Management and Analysis System (SORMAS), the latter was implemented in 2021 within a pilot scheme.

**Methods:**

We conducted interviews and a web-based survey distributed to users of the 3 surveillance tools. The survey assessed users’ views of the surveillance tools’ usefulness, ease of use, feelings toward the tool, conditions that may influence the use, and other characteristics. The descriptive analysis compared responses from SORMAS, MAGPI, and DHIS2 users, providing a comprehensive evaluation of their experiences.

**Results:**

Among the 159 respondents who actively use one of the systems, MAGPI was the most widely used surveillance tool among respondents (n=127, 79.9%), followed by DHIS2 (n=108, 67.9%), and SORMAS (n=25, 15.7%). In terms of users’ perceptions, SORMAS, despite its limited implementation, emerged as a tool that allows for data analysis and had the most comprehensive set of functionalities. DHIS2 was appreciated for its frequency of report provision, although users reported occasional IT system failures. MAGPI was recognized for its ease of use but was reported to lack certain functionalities offered by the other surveillance systems.

**Conclusions:**

This study offers valuable insights into the perceptions of disease surveillance tools users in Côte d’Ivoire. While all systems were positively regarded, each exhibited strengths and weaknesses addressing different needs and functionalities. Policy makers and health officials can use these findings to enhance existing tools or consider a unified approach for infectious disease surveillance systems. Understanding users’ perspectives allows them to optimize the choice of surveillance tools, ultimately strengthening public health responses in Côte d’Ivoire and potentially serving as a model for other countries facing similar decisions in their health care systems.

## Introduction

Accurate and reliable data are indispensable for planning and decision-making and for achieving our goal of a healthier world [1]. To accomplish this, well-functioning health information systems play a crucial role. These systems are designed to manage health care data by enabling data collection, storage, and sharing, and the support and operational management of health services and strategic decisions [2]. During outbreaks, epidemics, or pandemics such as the COVID-19 pandemic, surveillance systems that can monitor and manage emerging or re-emerging diseases have shown to be an essential tool for managing outbreaks. These systems, referred to as outbreak surveillance systems in this context, not only fulfill routine care functions but also provide close to real-time, accurate tracking, laboratory diagnostics, and notification capabilities to enable relevant stakeholders to understand the epidemiological situation and trends and to contribute to implement timely control measures [3,4].

In 2020, the Johns Hopkins University published a comprehensive report titled “Digital Solutions for COVID-19 Response: An assessment of digital tools for rapid scale-up for case management and contact tracing” [5]. This report extensively describes surveillance systems, evaluating their performance in various functionalities such as patient triage, referral for testing, contact tracing and notification, follow-up, and more. The 9 surveillance systems described in this report are the following: Surveillance Outbreak Response Management and Analysis System (SORMAS), District Health Information Software 2 (DHIS2), CommCare, Community Health Toolkit, Go.Data, Open Data Kit, Open Smart Register Platform, RapidPro, and WelTel. Additionally, the report analyzes 12 nonfunctional attributes of each system, such as usability, documentation, reliability, and scalability. Although the analysis highlights important technical attributes of surveillance systems, it does not explore the users’ views and experiences with these systems. Several authors have examined theories, such as the Unified Theory of Acceptance and Use of Technology (UTAUT), to understand users’ acceptance or rejection of new technologies [6-9]. These studies have revealed that factors such as usefulness, ease of use, and complexity significantly influence users’ decisions to adopt or reject a new technology.

In Côte d’Ivoire, the SORMAS was piloted in 2 regions (Abidjan and Gbêkê) and 11 districts, during July and August 2021 until June 2023. SORMAS is an open-source mobile and web application designed to enable health care workers and surveillance managers to promptly notify health services, policy makers, and other stakeholders about new cases of infectious diseases such as COVID-19, thereby facilitating an informed response. This pilot was carried out in the context of the “COVID-19-Outbreak Response combining E-health, Serolomics, Modelling, Artificial Intelligence, and Implementation Research” (CORESMA) project, which aims to generate urgently needed epidemiological data for defining targeted public health measures at national and global levels, effectively addressing the recent COVID-19 outbreak.

In many countries, including Côte d’Ivoire, it is observed that several infectious disease surveillance systems are concurrently in place [10]. During the baseline assessment survey of the CORESMA project, which was conducted in 11 districts of Côte d’Ivoire [11], we identified the use of DHIS2 and a surveillance tool by the company MAGPI, in addition to SORMAS. In such contexts, where multiple surveillance systems serve the same function, it is crucial to determine which system best fits the specific context and meets the needs of local users. The success of surveillance systems in achieving their goals implicitly depends on the acceptance or rejection of these systems by users, for instance, as well as factors such as political support [12].

To our knowledge, there has been no in-depth assessment among users of whether the outbreak surveillance systems used for emerging and re-emerging diseases in Côte d’Ivoire meet users’ expectations. Hence, our study aimed to first conduct a situational analysis of currently used outbreak surveillance systems and their key characteristics and, second, explore the users’ perceptions of these different systems.

## Methods

### Study Design and Setting

This cross-sectional study was conducted in Côte d’Ivoire, as part of the CORESMA project [13]. We first conducted exploratory discussions with resource persons closely involved in the implementation or the use of the different surveillance systems used for infectious disease surveillance (or both). This was followed by inviting stakeholders representing various groups in the health care sector to respond to a web-based questionnaire that assessed their usage and views of the available surveillance systems in Côte d’Ivoire, specifically examining whether these systems met their expectations concerning certain attributes. The selection of attributes was inspired by the UTAUT model [6] and the Johns Hopkins University report [5], as described in more detail below. Both served as frameworks that were then tailored to our specific study setting and objectives.

### Exploratory Interviews

In the first half of 2023, we conducted exploratory interviews with key individuals in Côte d’Ivoire who had direct involvement with the various surveillance systems to gain insights into several key aspects of the outbreak surveillance tools, such as local, regional, and national data managers. These discussions were guided by a series of predetermined questions. For each system, we learned about the system managers, the extent of geographical coverage, the specific diseases being surveilled, the flow of information and data, the approaches to data analysis, and the range of tasks supported by each system, among other relevant details.

### Web-Based Survey

#### Study Population and Sample Size

The web-based survey targeted health care professionals and decision makers using regularly at least one of the COVID-19 surveillance systems. Potential participants included all people responsible for managing infectious disease data for epidemiological surveillance at the district, regional, and national levels from all districts; these could be nurses, doctors, laboratory technicians, and medical record officers, among others. The target was to gather a minimum of 40 responses for each surveillance system, with 30 from health facility staff and 10 from surveillance managers (district, regional, and national levels). Considering an estimated response rate of around 15% (after reminders), we planned a minimum of 250 invitations sent to increase the chances of obtaining at least 40 responses for each surveillance system. The goal was to include staff members from a minimum of 3 different health facilities, with 40 respondents per system distributed accordingly. Participants who had used multiple systems were requested to provide feedback on all systems they have been using.

All potential participants received an email from the deputy director of the Institut National d’Hygiène Publique (INHP, [French: National Institute of Public Hygiene]) in Côte d’Ivoire, inviting them to collaborate by completing the questionnaire. This was a closed survey. Four email reminders were sent by the local partners followed by, if necessary, phone calls.

#### Structure of the Web-Based Questionnaire

This semistructured web-based questionnaire was designed to gather an overview of user perceptions regarding each surveillance system at the health facility, district, regional, and national level.

The web-based questionnaire was deployed using EvaSys (version 9.0; Electric Paper Evaluationssysteme GmbH). It consisted of 3 main sections (Multimedia Appendix 1). In the first section, respondents were asked about their sociodemographic information while ensuring questionnaire anonymity. They were also queried about the systems with which they were most familiar, as subsequent questions would pertain only to those specific systems.

The second section focused on participants’ perceptions of various attributes and factors related to the systems they used. A 5-point satisfaction Likert scale was used for responses. The questions drew mainly from the UTAUT model [6] and the Johns Hopkins University report [5]. From the UTAUT model, we included questions related to performance expectancy (usefulness), effort expectancy (ease of use and complexity), attitude toward using technology, and facilitating conditions. The Johns Hopkins University report influenced the inclusion of questions on documentation, reliability, devices and operating systems, and security.

The final section of the questionnaire encompassed questions regarding respondents’ overall opinions and satisfaction with the surveillance systems. Participants were asked to identify the factors most important to them in a surveillance system and indicate which systems they were most satisfied with. Additionally, comment boxes were provided throughout the questionnaire for any additional feedback or comments.

The questionnaire was translated to French. It was subsequently pretested, and necessary adjustments were made before deployment to the participants.

### Data Analysis

Data collected through the web-based questionnaire was descriptively analyzed by summarizing results such as frequencies and measures of central tendency. The number of people using each of the 3 surveillance tools for different surveillance-related tasks was compared using separate 3×2 chi-square tests, 1 for each task, with a predetermined significance level of *α*=.05. Similarly, Fisher exact tests were performed. Finally, we carried out a descriptive side-by-side comparison of participants’ views on the different outbreak surveillance systems. All data cleaning and analysis were performed using Stata (version 16; StataCorp).

### Ethical Considerations

This study was approved by the National Ethics Committee of Life Sciences and Health (*Comité National d’Ethique des Science de la Vie et de la Santé*) in Côte d’Ivoire (004‐23/MSHPCMU/CNESVS-km), and by the ethics committee of Northern and Central Switzerland (no AO 2022‐00070). Prior to entering the web-based questionnaire, all potential respondents were presented with information concerning this study and were asked whether they consented to participate. Participation was voluntary and no financial incentives or compensation was offered for participation. No names or any other identifiable information was collected ensuring participant anonymity.

## Results

### Desk Review and Exploratory Interviews

A total of 5 exploratory interviews were conducted in June 2023 to obtain information on key characteristics of each of the identified COVID-19 surveillance tools. Interviewees were users of the 3 surveillance tools who were familiar with the surveillance systems and their processes. The interview on SORMAS was conducted by author MSP jointly with the computer engineer who is responsible for the management of SORMAS in Côte d’Ivoire; the interviews concerning MAGPI were conducted with the participation of the statistician or data manager responsible for managing MAGPI in Côte d’Ivoire, as well as with country’s focal point for the flu; and the 2 interviews on DHIS2 were conducted with 2 epidemiological surveillance officers, in addition to the inputs provided by the statistician or data manager.

Table 1 summarizes the key characteristics of the 3 surveillance systems. We found that not all tools are overseen and administered by the same government institutions. SORMAS is the only tool that is not yet implemented in all districts of the country. There is a large overlap in the diseases surveilled. Compared to the other 2 systems, SORMAS tracks a lower number of emerging and re-emerging diseases. Although all tools are designed to allow for interoperability across systems, there is no data exchange and consolidation presently in place. Finally, our findings show that SORMAS is the only tool in which users can complete all the disease surveillance tasks we inquired about, which are listed in Table 1.

**Table 1.** Characteristics and functionalities reported during the interviews with key individuals in Côte d’Ivoire.

		DHIS2^a^	MAGPI	SORMAS^b^
Responsible for tool		Direction de l’Informatique et de l’Information Sanitaire (DIIS) an entity of the Ivory Coast Ministry of Health, Public Hygiene and Universal Health Coverage	INHP^c^, an affiliated institution of the Ivory Coast Ministry of Health, Public Hygiene and Universal Health Coverage	INHP an affiliated institution of the Ministry of Health, Public Hygiene and Universal Health Coverage
Geographical reach		All 33 administrative sanitary regions and all 113 districts	All 33 administrative regions and all 113 districts	2 administrative regions and 11 districts
Surveilled diseases		Yellow fever, dengue, cholera, measles, viral meningitis, bacterial meningitis, neonatal tetanus, guinea worm disease, diphtheria, hemorrhagic fevers, and acute poliomyelitis	COVID-19, yellow fever, dengue, cholera, measles, meningitis (viral and bacterial), neonatal tetanus, guinea worm disease, diphtheria, viral hemorrhagic fevers, acute poliomyelitis, flu, acute flaccid paralysis, and maternal deaths	COVID-19, yellow fever, dengue, cholera, measles, meningitis (viral and bacterial), neonatal tetanus, guinea worm disease, Ebola, flu, acute flaccid paralysis, and maternal deaths
Interoperability^d^		—^e^	—	—
Data analysis		Data are extracted to an Excel (Microsoft Corp) for analysis	MAGPI allows for basic analysis (eg, number of cases). Data are exported to Excel or Epi Info (Centers for Disease Control and Prevention) for analysis	Analysis is mostly carried out in SORMAS, and there is a form of aggregate data per week for each disease
Reports		Automatically generated reports	Reports generated by INHP (not automatically)	Automatically generated reports
**Which tasks does the system allow for?**				
	Contact tracing	Yes	No	Yes
	Capturing symptoms	Yes	Yes	Yes
	Entering demographic data	Yes	Yes	Yes
	Entering risk factor and exposure data	Yes	Yes	Yes
	Direct input of laboratory results	No	Yes (but not used)	Yes (but not used)
	Recording laboratory data	Yes	Yes	Yes
	Linking confirmed cases with contacts	Yes (for COVID-19)	No	Yes
	Monitor patient outcomes	Yes	Yes	Yes
	Manage cases (isolation checks)	Yes	No	Yes
	Ports of entry screening and follow-up	Yes	No	Yes

a DHIS2: District Health Information Software 2.

b SORMAS: Surveillance Outbreak Response Management and Analysis System.

c INHP: Institut National d’Hygiène Publique.

d In theory, all systems would be interoperable among each other, but this is not the case in real life.

e Not applicable.

**Figure 1.**
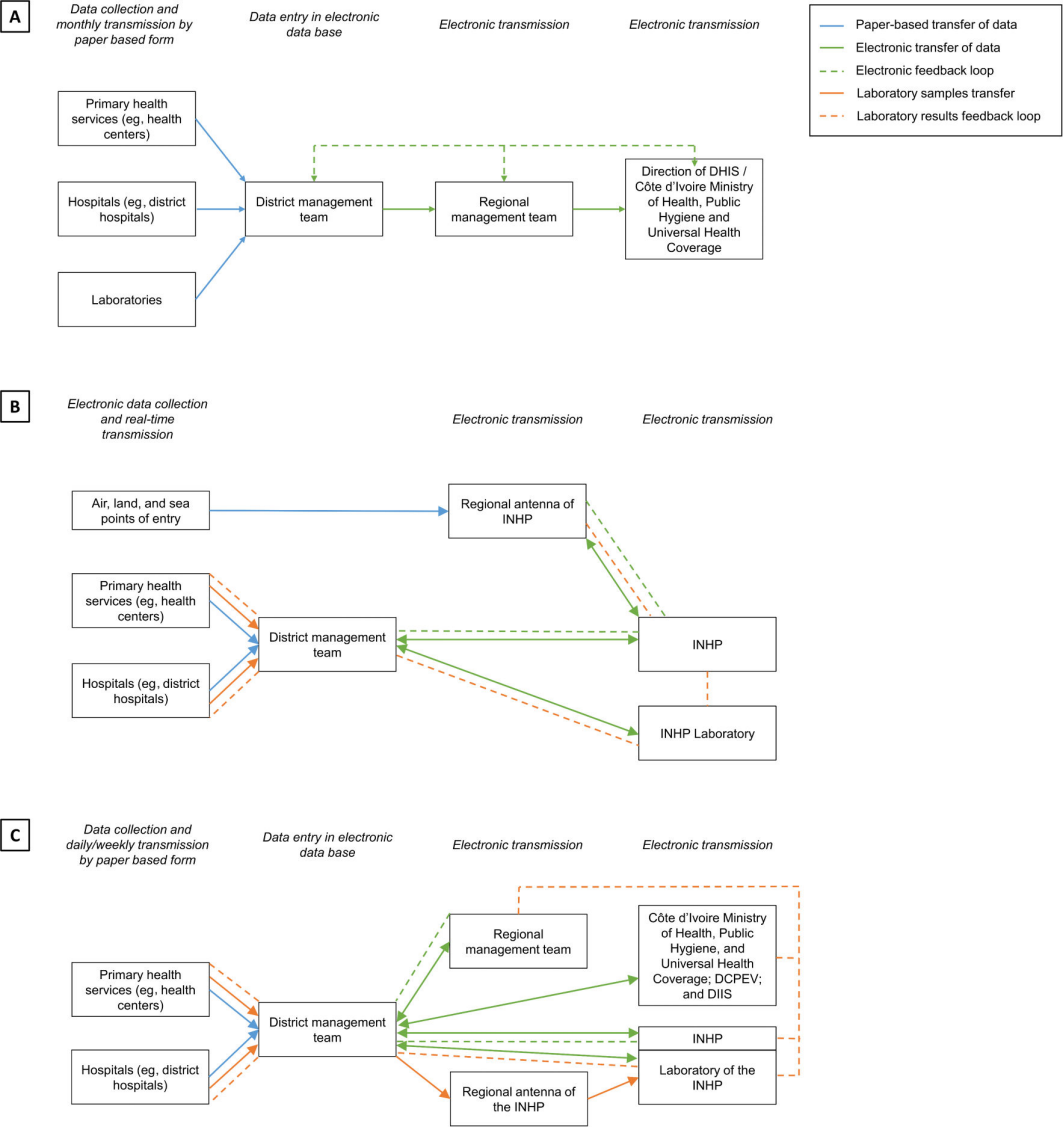
Flow of surveillance data using the (A) District Health Information Software 2 (DHIS2), (B) Surveillance Outbreak Response Management and Analysis System (SORMAS), and (C) MAGPI in Côte d’Ivoire. DCPEV: Direction de Coordination du Programme Elargi de Vaccination; DHIS: District Health Information Software; DIIS: Direction de l’Informatique et de l’Information Sanitaire; INHP: Institut National d’Hygiène Publique.

The interviews also allowed us to understand the data flow for each of the surveillance tools (Figure 1). The flow of information using DHIS2 and MAGPI still resorts to paper forms, whereas SORMAS is fully electronic. SORMAS also allows bidirectional flow of information to all entities involved. MAGPI is the only surveillance system in Côte d’Ivoire that is currently linked with the laboratories where samples are analyzed; SORMAS has this functionality as well but it was not being used.

### Web-Based Survey

Data were collected using the web-based questionnaire from March 13 to June 26, 2023. While the intention was to include 250 persons involved in managing the surveillance tools, 232 people potentially using at least one of the tools in Côte d’Ivoire could be identified. Of those, 207 individuals accessed the web-based questionnaire. Two declined to participate (they did not provide consent), resulting in a total of 205 respondents; this corresponds to a response rate of 88.4%. However, of the 205 participants who started responding to the questionnaire, 46 had never used any of the three tools and, therefore, did not provide responses concerning the surveillance systems. These were found to be people who were trained in one of the 3 systems but did not actively use any of them afterward. Thus, they were not further included in the analysis so that the responses of a total of 159 participants were considered. Because some respondents use more than 1 system, 130 responses on MAGPI, 117 on DHIS2, and 29 on SORMAS among the 159 respondents could be analyzed. Table 2 presents the demographic characteristics of the respondents. Most respondents were male (n= 124, 78.0%) and the 2 most prevalent age groups were 41-50 years (n= 62, 39.0%) followed by 31-40 years (n= 59, 37.1%).

**Table 2.** Characteristics of the participants of the web-based questionnaire (n=159).

Characteristics		Participants, n (%)
**Sex**		
	Female	28 (17.6)
	Male	124 (78.0)
	No answer	7 (4.4)
**Age (years)**		
	18-30	10 (6.3)
	31-40	59 (37.1)
	41-50	62 (39.0)
	51-60	24 (15.1)
	>60	5 (0.6)
	No answer	3 (1.9)
**Profession or function** ^a^		
	Data manager	48 (30.2)
	Epidemiology surveillance officer	108 (67.9)
	Nurse	77 (48.4)
	Midwife	7 (4.4)
	Pharmacist	2 (1.3)
	Doctor	5 (2.4)
	Laboratory technician	1 (0.5)
	Health care unit supervisor	1 (0.5)
	Surveillance officer (national, regional, or district level)	29 (14.1)
	Other	21 (13.2)

a More than one selection is possible given that respondents might have a profession (eg, nurse) as well as a specific function (eg, data manager).

**Figure 2.**
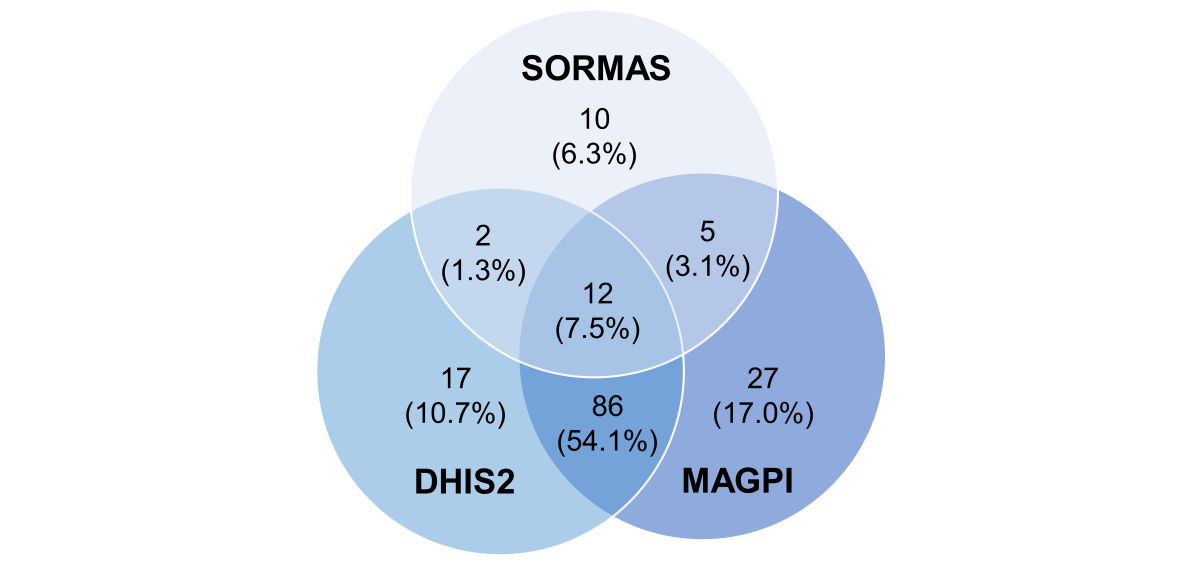
Venn diagram depicting number of participants who are current or past users of surveillance tools and nonusers. DHIS2: District Health Information Software 2; SORMAS: Surveillance Outbreak Response Management and Analysis System.

Given that we aimed at collecting the users’ views on each of the 3 surveillance systems, we included participants who either use a tool at the time of survey (first quarter 2023) or have used it in the past, as this indicates their familiarity with the tool. MAGPI was found to be the most widely used surveillance tool among respondents (n=127, 79.9%), followed by DHIS2 (n=108, 67.9%), and SORMAS (n=25, 15.7%; Table 3). The lower user adoption of SORMAS is not surprising because unlike MAGPI and DHIS2 that have been established as national-scale tools in 2013, SORMAS was introduced through a pilot in 2021 in 2 regions (urban areas of Abidjan and 2 sites in rural Gbêkê). In total, 12 (7.5%) participants have experience with all 3 tools and 98 (61.6%) had experience with both MAGPI and DHIS2 (Figure 2). As mentioned, 46 respondents had never used any of the 3 tools.

**Table 3.** Usage patterns of each of the surveillance tools.

Usage patterns				Participants, n (%)			*P* value
				MAGPI	DHIS2^a^	SORMAS^b^	
Currently uses it				127 (79.9)	108 (67.9)	25 (15.7)	N/A^c^
Used it in the past but not now				3 (1.9)	9 (5.7)	4 (2.5)	N/A
Has never used it				29 (18.2)	42 (26.4)	130 (81.8)	N/A
**Frequency of use** ^d^							
	Several times per day			55 (43.3)	67 (62.0)	8 (32.0)	N/A
	Once per day			7 (5.5)	4 (3.7)	2 (8.0)	N/A
	2 to 3 times per week			35 (27.6)	16 (14.8)	4 (16.0)	N/A
	Once per week			26 (20.5)	7 (6.5)	5 (20.0)	N/A
	Every 2 weeks			1 (0.8)	1 (0.9)	0 (0.0)	N/A
	Once per month			2 (1.6)	11 (10.2)	1 (4.0)	N/A
	Less than once per month			1 (0.8)	2 (1.9)	5 (20.0)	N/A
**Tasks the surveillance tool is used for** ^e^							
	Case detection and management			71 (54.6)	16 (22.2)	16 (55.2)	<.001
	Contact registration and follow-up			58 (44.6)	17 (14.5)	22 (75.9)	<.001
	Port of entry screening and follow-up			8 (6.2)	5 (4.3)	15 (51.7)	<.001
	Facility readiness and stock tracking			2 (1.5)	2 (1.7)	0 (0.0)	.79
	Health care worker training and monitoring			12 (9.2)	17 (14.5)	2 (6.9)	.39
	Entry and follow-up of laboratory tests			27 (20.8)	4 (3.4)	13 (44.8)	<.001
	Event-based surveillance			77 (59.2)	31 (26.5)	14 (48.3)	.005
	Reporting			69 (53.1)	80 (68.4)	15 (51.7)	.43
	Informing patients			13 (10.0)	5 (4.3)	4 (13.8)	.16
	Clinical management of cases			19 (14.6)	17 (14.5)	7 (24.1)	.54
	Others			—^f^	28 (23.9)	—^f^	N/A
**Other usage questions (yes/no questions)**							
	In their opinion, this system be used on its own to manage COVID-19, for example, without using additional systems			81 (62.3)	76 (65.0)	22 (75.9)	N/A
**Data analysis**			
	Data are exported to Excel (Microsoft Corp) for data analysis			116 (89.2)	102 (87.2)	12 (41.4)	N/A	
	Data analysis is conducted on with surveillance tool			9 (6.9)	15 (12.8)	17 (58.6)	N/A	
	Data are analyzed in another format			5 (3.8)	0 (0.0)	0 (0.0)	N/A	
	Data exportation is easy and works well			115 (88.5)	106 (90.6)	19 (65.5)	N/A
	System provides regular consolidated feedback information (such as monthly reports)			78 (60.0)	98 (83.8)	20 (69.0)	N/A

a DHIS2: District Health Information Software 2.

b SORMAS: Surveillance Outbreak Response Management and Analysis System.

c N/A: not applicable.

d Only asked to those who currently use the surveillance tool.

e Due to an error in the web-based questionnaire, the option of “other” was not provided to MAGPI and SORMAS users.

f Not available.

The majority of users reported using the surveillance tools every day (Table 3). When considering the specific tasks for which these tools are used, SORMAS stood out as the most commonly used tool in all categories except for “facility readiness and stock tracking,” “event-based surveillance,” and “reporting.” Particularly notable differences were observed in the tasks of “contact registration and follow-up,” “port of entry screening and follow-up,” and “entry and follow-up of laboratory tests,” where SORMAS was used considerably more often than MAGPI and DHIS2. Furthermore, SORMAS users expressed the highest level of agreement (n=22, 75.9%) that this tool can be used independently without the need for additional systems, followed by DHIS2 (n=76, 65.0%) and MAGPI (n=81, 62.3%).

DHIS2 users indicated that they are missing the DHIS2 Tracker, that the DHIS2 does not allow for real-time notifications, and that data are only available 24 hours after being inputted, among others.

Regarding data analysis, the majority of MAGPI (n=116, 89.2%) and DHIS2 (n=102, 87.2%) users reported exporting the data to an Excel (Microsoft Corp) spreadsheet for analysis (Table 3). In contrast, only 41.4% (n=12) of SORMAS users engaged in data exportation for analysis, with most SORMAS users (n=17, 58.6%) conducting their data analysis directly within the SORMAS platform. However, a lower proportion of SORMAS users (n=19, 65.5%) expressed satisfaction with the ease and functionality of exporting their data (when necessary), compared to MAGPI (n=115, 88.5%) and DHIS2 (n=106, 90.6%) users.

In terms of feedback provided by the surveillance tools, such as regular reports, DHIS2 was indicating as offering the most frequent reports (n=98, 83.8%), followed by SORMAS (n=20, 69.0%), and finally MAGPI (n=78, 60.0%; Table 3).

The chi-square tests (with detailed Fisher exact test results provided in Multimedia Appendix 2) revealed specific tasks where DHIS2 exhibited significantly lower usage than MAGPI and SORMAS (Table 3). Furthermore, an overarching trend suggests that SORMAS encompasses a broader spectrum of tasks overall.

**Figure 3.**
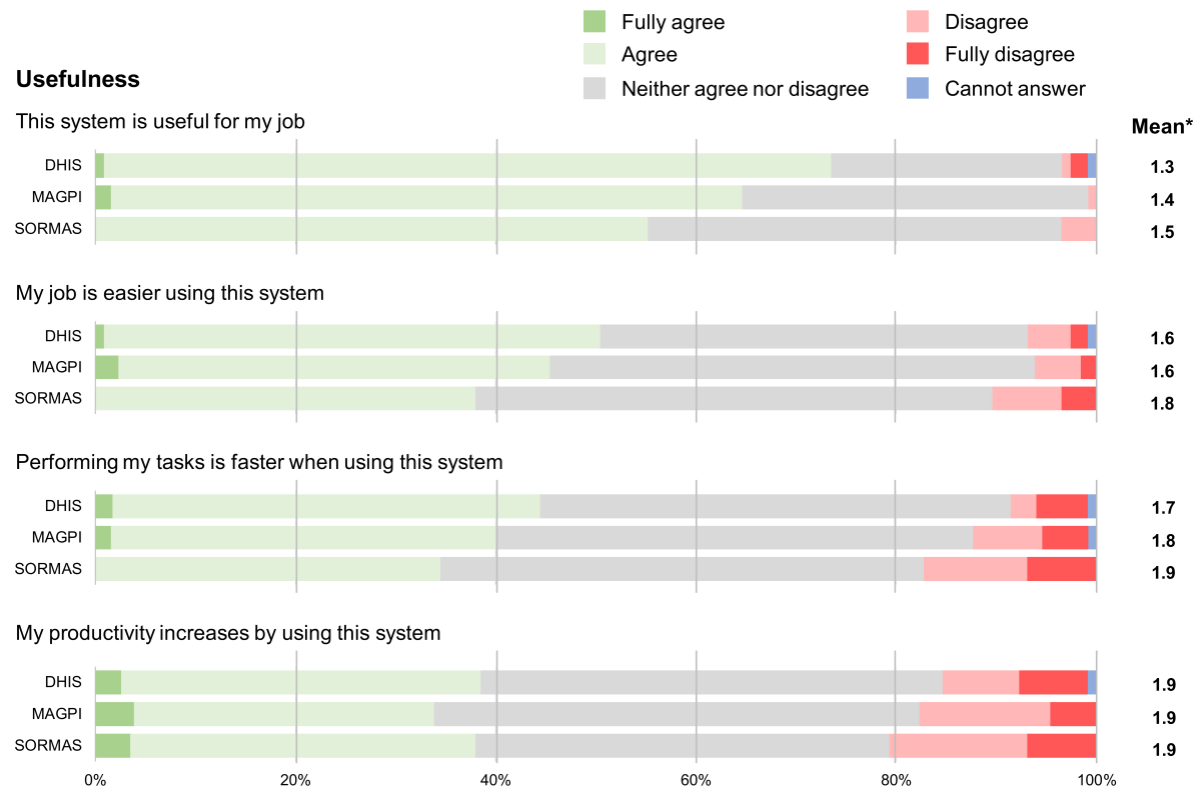
Likert scale results of the users’ perception for each of the statements related to the usefulness of the 3 different surveillance tools. *Mean of the Likert scale results excluding “Cannot answer” responses and where 1=fully agree and 5=fully disagree. DHIS: District Health Information Software; SORMAS: Surveillance Outbreak Response Management and Analysis System.

The following figures show the Likert scale results of the users’ perceptions for each question pertaining to the tool’s usefulness, ease of use, feeling toward it, conditions that may influence the use of the tool, and other tool characteristics. Additionally, they also show the mean of the Likert scale results (1=fully agree and 5=fully disagree). Concerning its usefulness, SORMAS tended to have fewer good results (Figure 3). Despite all tools resulting in similar findings, a slightly lower proportion of people using SORMAS agreed that it was useful for their job, made their job easier and made the performance of their tasks easier.

**Figure 4.**
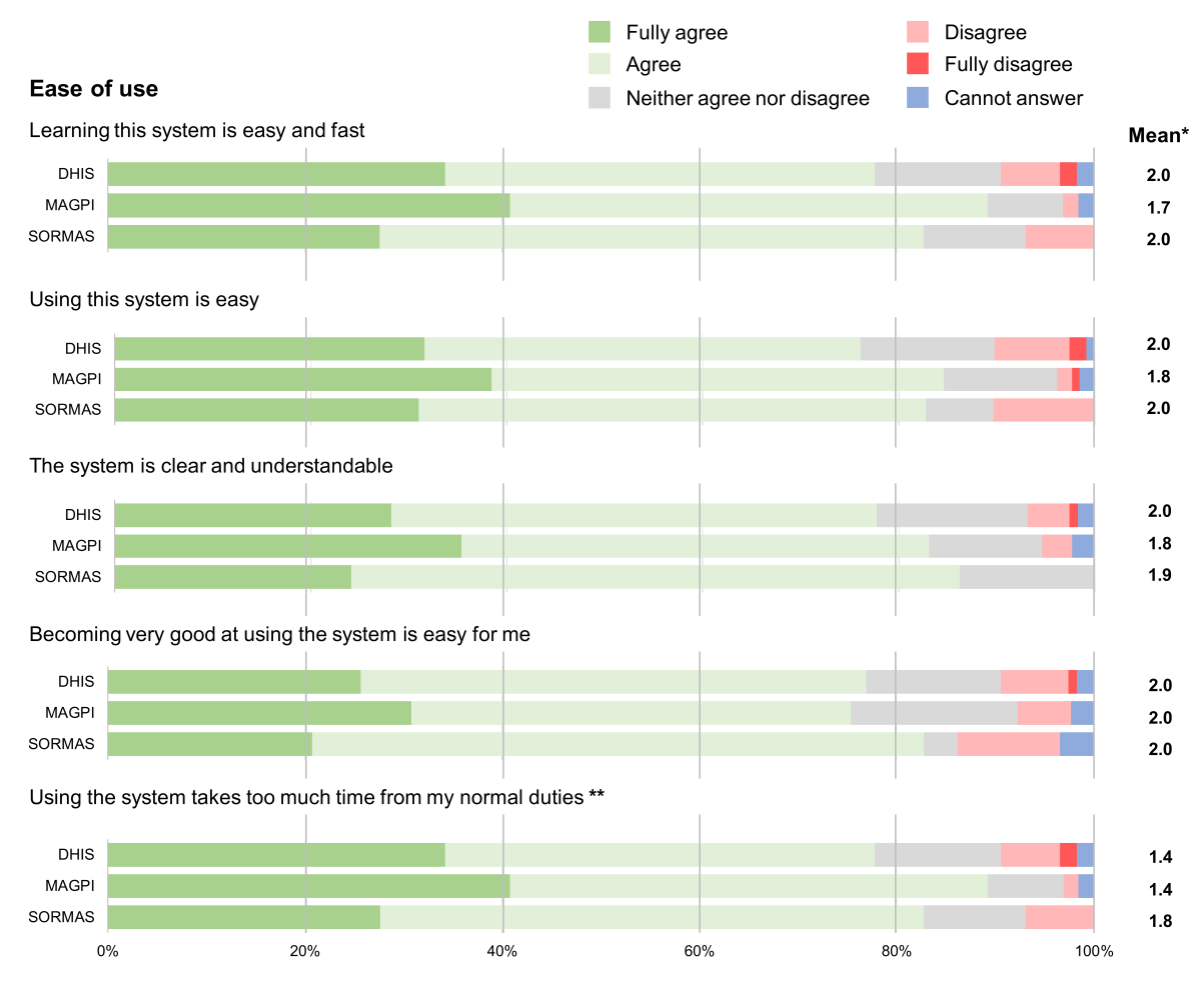
Likert scale results on the users’ perception for each of the statements related to the ease of use of the three different surveillance tools (DHIS, MAGPI and SORMAS). *Mean of the Likert scale results excluding “Cannot answer” responses and where 1=fully agree and 5=fully disagree. **Negative affirmation was used, so the score was inverted for the purpose of this figure.

Regarding the ease of use of these surveillance systems, there appears a clear trend where MAGPI and its tools were considered the easiest to use, followed by DHIS2 and then SORMAS; however, the differences are comparatively small (Figure 4).

**Figure 5.**
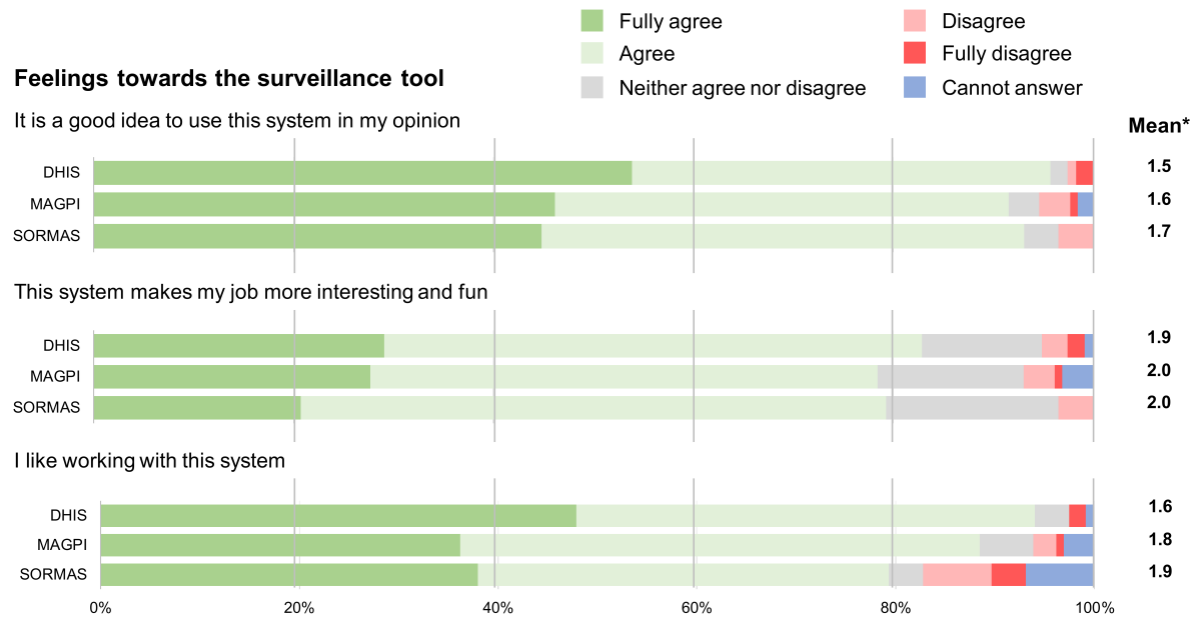
Likert scale results on the users’ perception for each of the statements related to the feelings toward the 3 different surveillance tools (DHIS [District Health Information Software], MAGPI, and SORMAS [Surveillance Outbreak Response Management and Analysis System]). *Mean of the Likert scale results excluding “Cannot answer” responses and where 1=fully agree and 5=fully disagree.

We found that participants’ feelings toward each tool was mostly very positive, with almost every person agreeing it was a good idea to use any of the 3 tools (Figure 5). SORMAS had fewer positive results than the other tools, when it came to whether people like to work with the system or not.

**Figure 6.**
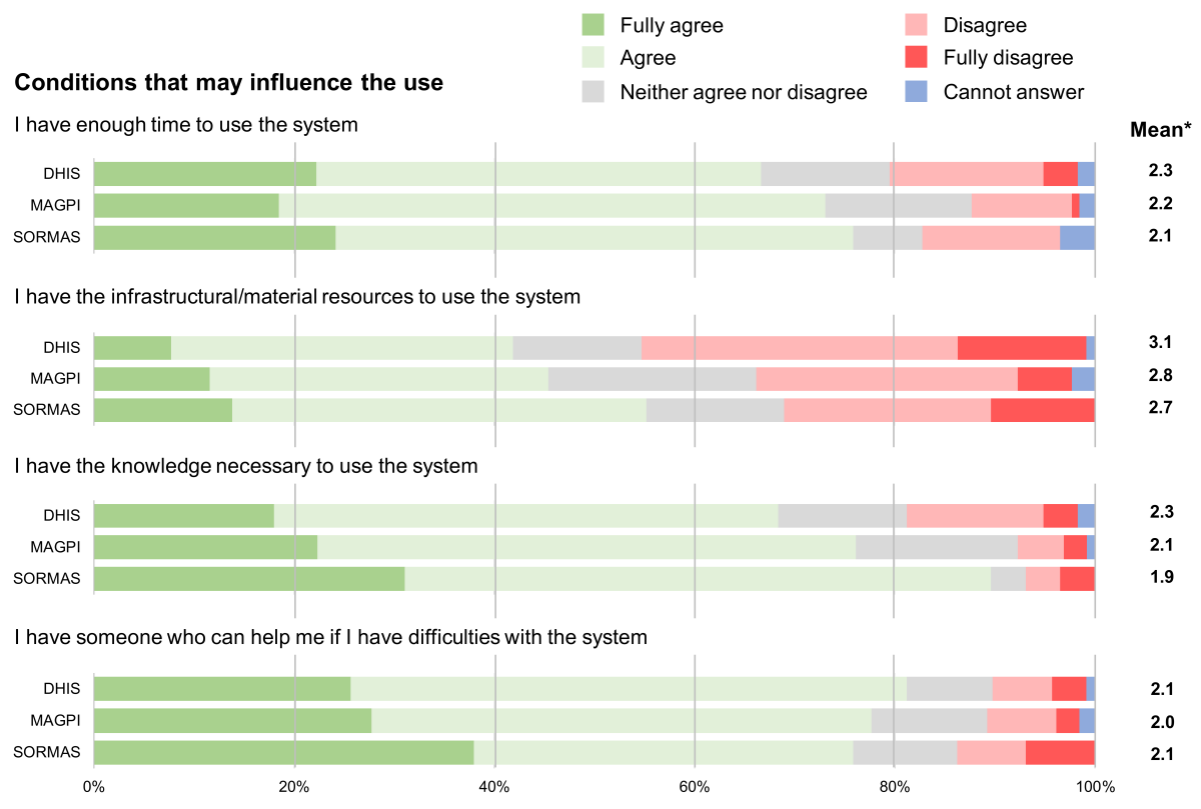
Likert scale results on the users’ perceptions for each of the statements related to the conditions that may influence the use of the three different surveillance tools (DHIS [District Health Information Software], MAGPI and SORMAS [Surveillance Outbreak Response Management and Analysis System]). *Mean of the Likert scale results excluding “Cannot answer” responses and where 1=fully agree and 5=fully disagree.

In terms of conditions that may influence the use of these surveillance systems, our results point toward a more negative perception, compared to the other attributes (Figure 6). A large proportion of the participants disagreed having the infrastructural or material resources to use the tool, especially DHIS2, followed by MAGPI and SORMAS. Additionally, about one-fifth of DHIS2 users reported to not have sufficient time and knowledge to use the tool.

**Figure 7.**
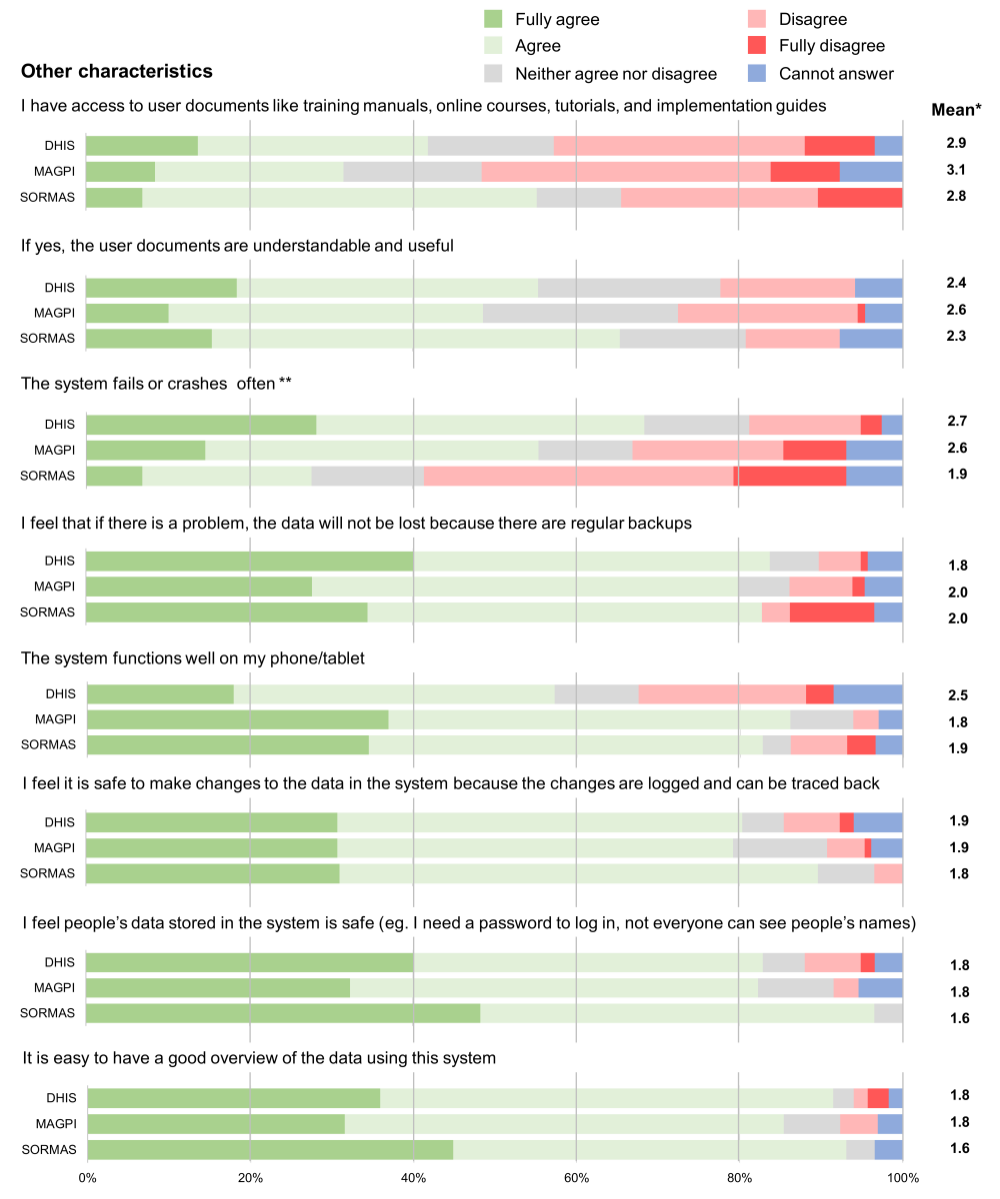
Likert scale results on the users’ perception for each of the statements related to other characteristics of the 3 different surveillance tools (DHIS [District Health Information Software], MAGPI, and SORMAS [Surveillance Outbreak Response Management and Analysis System]). *Mean of the Likert scale results excluding “Cannot answer” responses and where 1=fully agree and 5=fully disagree. **Negative affirmation was used, so the score was inverted for the purpose of this figure.

Concerning a few other characteristics that did not fall into any of the previous attributes, we found that a large proportion of users, irrespective of which tool they use, do not have access to training materials and guidelines (Figure 7). Those who do have access, did not necessarily agree they are easy to access, understandable, and useful.

Over half of SORMAS users agree that the system is more stable as it does not fail often, whereas DHIS and MAGPI users agree that it often fails. DHIS2 users were the least likely to agree that the system works well on their phone or tablet. In terms of feeling it is safe to make changes to the data and that the data itself is safely stored, all 3 systems had similar results.

## Discussion

### Principal Results and Comparison With Prior Work

Many countries use, in parallel, multiple surveillance systems and tools for identifying and monitoring infectious disease outbreaks [10]. In many countries, the COVID-19 pandemic has led to strengthening surveillance or to the testing and roll-out of a new surveillance system (or both) as this was the case in Côte d’Ivoire through the piloting of SORMAS in 2 regions. However, this practice leads to inefficiencies and ineffectiveness in health data management. Aspects such as fragmentation of data collection, management, analysis, and reporting are a concern [14]. In this context, we undertook this study to comprehensively evaluate users’ views and experiences with the 3 surveillance systems being used in Côte d’Ivoire. The insights gained from this study can offer valuable guidance to policy makers contemplating the adoption of a unified surveillance systems, aiding them in making informed decisions regarding potential transitions to a singular system meeting all or at least most of their needs.

Overall, we found that the 3 systems were predominantly positively perceived by their users. However, there were slight variations in perceptions with experienced advantages and drawbacks to each tool.

SORMAS was reported to operate more stably with fewer system failures and was found to score highest when it came to conditions that influence its use, such as availability of infrastructure and material, knowledge concerning the tool, and having access to support in case of any issues. The fact that SORMAS users reported more often to have the necessary conditions, could be related to its recent implementation as a pilot and that, in addition, this implementation was closely supervised by INHP. Another significant finding was that 58.6% (n=17) of SORMAS users perform data analysis on the system itself, which is substantially higher than that reported for MAGPI (n=9, 6.9%) and DHIS2 (n=15, 12.8%). This is advantageous since a system is only as good as users perceive a direct benefit from it.

Furthermore, our findings revealed that SORMAS allowed for the completion of a greater number of disease surveillance-related tasks compared to the other two surveillance systems. This is in line with findings from a recent study in Ghana investigating facilitators and barriers encountered during the implementation of SORMAS in the country, which reported relative advantages for task performance with SORMAS, including real-time reporting and the integration of laboratory procedures [15]. The John Hopkins report also concluded that SORMAS has the majority of the functions that decision makers identified as important for primary use case [5].

However, there were also disadvantages to SORMAS. For instance, SORMAS users were the ones to report most often (n=8, 34.5%) that the data exportation did not work well, emphasizing the need for improvements in this area. Additionally, SORMAS performed least well on all questions related to perception of usefulness. It is important to note that SORMAS was introduced last and in parallel with the existing tools, which led to duplicated efforts, likely contributing to lower perceived usefulness scores. This led to a doubling of the effort to enter data and could explain some of our findings.

MAGPI and DHIS2 are both very well-established disease surveillance tools, which have been deployed to all regions and districts of Côte d’Ivoire since 2013. Our findings suggest that DHIS2 is more complete in terms of tasks it allows than MAGPI, and provides the most frequent regular reports. Also, the study found that users have more positive feelings toward DHIS2 than toward the other tools. However, DHIS2 was reported to be the system that encounters errors and unexpectedly terminates most often. In terms of ease of use, MAGPI scored highest in all questions.

These findings suggest that none of the 3 surveillance tools cover all the needs for the effective control of infectious diseases, as they all have drawbacks. However, it is important to note as well that, as important as the choice of the surveillance tool is, it is necessary to note that a strong surveillance system alone is not sufficient. A recent study found that inadequate trained human resources, poor infrastructure, and coordination challenges can hamper the effective implementation of surveillance tools [16]. The study in Ghana also reported barriers to the implementation of SORMAS such as unstable national power supply, and substantial dependence on external funding, for example.

These findings indicate that none of the 3 surveillance tools fully meet the all the needs for the effective control of infectious diseases, as each has its limitations. It is crucial to emphasize that, while the choice of the surveillance tool is very important, a robust surveillance system alone does not guarantee success. A recent study highlighted that factors like insufficiently trained human resources, poor infrastructure, and coordination challenges can hamper the effective implementation of surveillance tools [16]. Similarly, in Ghana, several barriers in the implementing SORMAS were reported, including an unstable national power supply and substantial reliance on external funding [15]. Therefore, addressing these potential barriers is critical alongside the selection of a comprehensive surveillance tool.

A complementary finding of this study indicated by the remarkably high response rate we obtained (89%) was the potential of web-based surveys as a valuable tool for cost-effective and streamlined data collection, at least in the context of the health system of Côte d’Ivoire.

### Limitations

This is the first study investigating the users’ perceptions of various disease surveillance tools in Côte d’Ivoire, and we were able to compare all surveillance tools used in the country for the surveillance of COVID-19. Ideally, we would have had a higher number of SORMAS users responding to the web-based survey; however, this was not feasible as the tool has been piloted in only 2 regions. Finally, as much as we attempted to use simple yet clear language in the questionnaire, it seems there may have been at least one question that was not quite fully understood by respondents the way we intended it to be. When we inquired about tasks completed using the different surveillance tools, our intention was to understand the tasks respondents themselves performed, not the tool’s capabilities. However, in this instance, we observed that some participants answered based on their knowledge of what SORMAS can do, especially regarding the task labeled “Entry and follow-up of laboratory tests”; 44.8% (n=13) of SORMAS respondents claimed to use it for this task, even though exploratory interviews with key individuals revealed that this particular functionality of SORMAS was not being used in Côte d’Ivoire at that time.

### Conclusions

To fulfil its role in the surveillance of infectious diseases, a surveillance system must embody various essential characteristics, such as reliability, scalability, and usability [5]. These attributes collectively contribute to a system’s efficacy and resilience. Nevertheless, the effectiveness of any surveillance system depends on its use, a factor directly influenced by the perceptions of its users.

This comprehensive study delved into the perceptions of professionals using 3 distinct surveillance tools—DHIS2, MAGPI, and SORMAS. We examined factors such as usefulness, ease of use, feelings toward the tool, conditions that may influence the use, among others. Our findings showed that each system had its own strengths and weaknesses, and none of them singularly addressed all the needs.

In our perspective, for a surveillance system to be truly effective, a unified approach emerges as the optimal strategy, likely built upon a single, robust surveillance tool. Although each system showed strengths, the gaps identified and differing strengths highlight the advantages of consolidating efforts into one comprehensive system that can serve as the backbone of a nation’s infectious disease surveillance infrastructure. However, considering the changing nature of infectious diseases, one needs to be always open to integrating complementary tools when necessary.

## Supplementary material

10.2196/56275 Multimedia Appendix 1 Questionnaire used for the web-based survey.

10.2196/56275 Multimedia Appendix 2 Number and proportion of participants using each of the 3 surveillance tools for different surveillance-related tasks, with associated *P* values from the Fisher exact tests.

## Abbreviations

CORESMA: COVID-19-Outbreak Response combining E-health, Serolomics, Modelling, Artificial Intelligence, and Implementation Research
DHIS2: District Health Information Software 2
INHP: Institut National d’Hygiène Publique
SORMAS: Surveillance Outbreak Response Management and Analysis System
UTAUT: Unified Theory of Acceptance and Use of Technology
