# Supplementary material for: Key Characteristics and Perception of Different Outbreak Surveillance Systems in Côte d’Ivoire: Cross-Sectional Survey Among Users
Source: JMIR Public Health Surveill. 2024 Jul 30;10:e56275. doi: 10.2196/56275 (PMC11300380; doi:10.2196/56275)
Supplement: Multimedia Appendix 1 [file publichealth-v10-e56275-s001.docx]

**Appendix 1.** Questionnaire used for online survey

**Name of study:** Digital tools for epidemic surveillance in Nepal and Côte d'Ivoire: main features and user perceptions

The implementation of this study has been validated by the Comité National d'Ethique des Sciences de la Vie et de la Santé in Côte d'Ivoire (acceptance reference: 004-23/MSHPCMU/CNESVS-km), reachable by email (contact@comite-ethique.fr) or by phone (01 42 75 66 42).

**What is the main objective of this study?** We want to explore your perceptions and opinions on how the existing epidemiological surveillance systems are working. This is because we want to use a new digital application (SORMAS) for epidemiological disease surveillance that meets the expectations of those involved in surveillance. With these results, we hope to contribute to optimizing and improving the surveillance system for all.

**Why do we invite you to take part in this study?** As someone who will be/is already directly involved in the implementation or use of the monitoring system, we believe that your opinion on this subject is of great value and will help us achieve the objectives of our study. Your participation is voluntary and there will be no negative consequences if you refuse to participate or withdraw at any stage of the study.

**What does your participation involve?** We ask you to complete a questionnaire in which we ask for your opinion of surveillance systems, as well as your age category, gender and job title. You will not be paid to participate.

**Will anyone know what you answered in the questionnaire?** Your answers are anonymous; only the investigators will have access to your personal information (gender, age category and job title); no identifiable data will be published in any form. Each participant's answers will be anonymized thanks to a system of individual codes for each participant. Participants' personal data will be destroyed at the end of the study by deleting them from all computers and servers where they were stored (the Institut National d'Hygiène Publique, Côte d'Ivoire, and the Swiss Tropical and Public Health Institute, Switzerland).

**What will the results of this questionnaire be used for?** The research team will analyze the responses of all respondents and prepare a manuscript for publication in a scientific journal to disseminate the results.

**This study is being carried out jointly by researchers from the following institutions:**

- Institut National d'Hygiène Publique, Côte d'Ivoire

- Dhulikhel Hospital Kathmandu University Hospital, Kathmandu University School of Medical Sciences, Nepal

- Swiss Tropical and Public Health Institute (Swiss TPH), Basel, Switzerland

If you have any questions, please do not hesitate to contact Dr Daouda Coulibaly at the Institut National d'Hygiène Publique by email (daocoul@yahoo.fr) or telephone (07984752/21259746).

If you answer "yes" to the question below, you confirm that you have read this information sheet, that you agree with what will be done during the study, that you understand how it will be done and what is asked of you. You also agree that you are aware that you can stop your participation at any time without inconvenience or explanation.

| **0. Do you agree to participate in this study?** | |
| --- | --- |
|  | Yes |
|  | No |

*If respondent consented to participate he/she would be redirected to answer questions 1 to 19. If not, he/she would be thanked and no further questions would be asked.*

| **1. What is your gender?** | |
| --- | --- |
|  | Female |
|  | Male |
|  | Other |
|  |  |
| **2. In which age group do you belong?** | |
|  | 18-30 years |
|  | 31-40 years |
|  | 41-50 years |
|  | 51-60 years |
|  | Older than 61 years |
|  |  |
| **3. What is your job?** | |
|  | *(You can select more than one option if your profession and role/function are different)* |
|  | Data manager |
|  | Epidemiology surveillance officer |
|  | Nurse |
|  | Midwife |
|  | Pharmacist |
|  | Doctor |
|  | Laboratory technician |
|  | Healthcare unit supervisor |
|  | Surveillance officer (national, regional or district level) |
|  | Data manager |
|  | Other |
|  |  |
| **5. Which disease surveillance system or systems do you have experience working with? Which ones do you currently use, which ones have you used in the past but no longer use and which ones have you never used?** | |
| *(Select one answer for each system listed below)* | |
|  | Select one answer for each of the systems listed below |
|  | DHIS2 (**only** select this option if you use it for **DISEASE SURVEILLANCE**) |
|  | EWARS |
|  | IMU |
|  | SORMAS |
|  | Excel |
|  |  |
|  | *(The next questions concern your use of* ***DHIS2****)*  *Questions 6 to 13 were asked for each of the surveillance systems respondents said they currently used or have used in the past.* |
| **6. How frequently do you use DHIS2?** | |
|  | Every day, several times per day |
|  | Once per day |
|  | 2-3 times per week |
|  | Once per week |
|  | Every two weeks |
|  | Once per month |
|  | Less than once per month |
|  |  |
| **7. For what tasks do you use DHIS2 for?** | |
| *(You can select more than one option if you need)* | |
|  | Case detection and management |
|  | Contact registration and follow-up |
|  | Port of entry screening and follow-up |
|  | Facility readiness and stock tracking |
|  | Healthcare worker training and monitoring |
|  | Laboratory test tracking |
|  | Event-based surveillance |
|  | Reporting |
|  | Informing patients |
|  | Clinical management of cases |
|  | Others |
|  |  |
|  | *(In the next questions, we ask you to share your opinions/perceptions of* ***DHIS2*** *concerning different aspects)*  *For questions 8 to 13 respondents were asked to rank each of the statements using the following Likert scale: fully agree, agree, neither agree not disagree, disagree, fully disagree.* |
| **8. Of these characteristics listed below, which 3 are most important for you in an outbreak surveillance system:** | |
|  | Whether I have access to good training documents and user instructions |
|  | How useful it is for my job by making it easier and faster |
|  | How easy to learn and use it is |
|  | Having the resources, the knowledge and the support to use the system |
|  | Whether I like using the system or not |
|  | How reliable the system is by not crashing, failing or losing data often |
|  |  |
| **9. Concerning the usefulness of this system, please let us know to what extent you agree or disagree with the statements below:** | |
|  | This system is useful for my job |
|  | My job is easier using this system |
|  | Performing my tasks is faster when using this system |
|  | My productivity increases by using this system |
|  |  |
| **10. Concerning the ease of use, please let us know to what extent you agree or disagree with the statements below:** | |
|  | Learning this system is easy and fast |
|  | Using this system is easy |
|  | The system is clear and understandable |
|  | Becoming very good at using the system is easy for me |
|  | Using the system takes too much time from my normal duties |
|  |  |
| **11. Concerning your feelings towards the system, please let us know to what extent you agree or disagree with the statements below:** | |
|  | It is a good idea to use this system in my opinion |
|  | This system makes my job more interesting and fun |
|  | I like working with this system |
|  |  |
| **12. Concerning conditions that may influence your use of the system, please let us know to what extent you agree or disagree with the statements below:** | |
|  | I have enough **time** to use the system |
|  | I have the **infrastructural/material resources** to use the system |
|  | I have the **knowledge** necessary to use the system |
|  | I have someone who can **help** **me** if I have **difficulties** with the system |
|  |  |
| **13. Concerning several other characteristics of the system, please let us know to what extent you agree or disagree with the statements below:** | |
|  | I have access to user documents like training manuals, online courses, tutorials, and implementation guides |
|  | The user documents are understandable and useful *(only if previous answer was yes)* |
|  | The system fails or crashes often |
|  | I feel that if there is a problem, the data will not be lost because there are regular backups |
|  | The system functions well on my phone/tablet |
|  | I feel it is safe to make changes to the data in the system because the changes are logged and can be traced back |
|  | I feel people’s data stored in the system is safe (eg. I need a password to log in, not everyone can see people’s names) |
|  | It is easy to have a good overview of the data using this system |
|  |  |
| **14. Of the system characteristics listed below, which three do you feel DHIS2 performs best at:** | |
| *(Please select 3)* | |
|  | Having access to good training documents and user instructions/support |
|  | Being useful for my job by making it easier and faster |
|  | Being easy to learn and use |
|  | Having the resources, the knowledge and the support to use the system |
|  | Liking to use the system |
|  | Being reliable by not crashing, failing or losing data often |
|  |  |
| **15. In your opinion, can this system be used on its own to do the management of COVID-19, for example, or would you need additional systems to do it?** | |
|  | Yes |
|  | No |
|  |  |
| **16. What functionalities are missing in this tool?** | |
|  | *(open answer)* |
|  |  |
| **17. Do you export the data from this tool into Excel format?** | |
|  | Yes |
|  | No, I do the analysis on DHIS2 |
|  | No, I perform the analysis in another format which is: |
|  |  |
| **18. Is it easy to export your data from this tool? Does the exportation work well?** | |
|  | Yes |
|  | No |
|  | **If not, why?** |
|  |  |
| **19. Is the system you use providing your regularly with consolidates feedback information (such as monthly reports for example)?** | |
|  | Yes |
|  | No |
|  |  |

*Respondents were thanked for their time and cooperation.*
