# Supplementary material for: Key Characteristics and Perception of Different Outbreak Surveillance Systems in Côte d’Ivoire: Cross-Sectional Survey Among Users
Source: JMIR Public Health Surveill. 2024 Jul 30;10:e56275. doi: 10.2196/56275 (PMC11300380; doi:10.2196/56275)
Supplement: Multimedia Appendix 2 [file publichealth-v10-e56275-s002.docx]

**Appendix 1.** Number of participants (%) using each of the three surveillance tools for different surveillance-related tasks, with associated p-values from Fisher’s exact tests.

|  | |  | MAGPI | DHIS2 | SORMAS |  |
| --- | --- | --- | --- | --- | --- | --- |
| **Tasks the surveillance tool is used for** | | | | | | **p-value** |
|  | Case detection and management | | 71 (54.6) | 16 (22.2) | 16 (55.2) | < 0.001 |
|  | Contact registration and follow-up | | 58 (44.6) | 17 (14.5) | 22 (75.9) | < 0.001 |
|  | Port of entry screening and follow-up | | 8 (6.2) | 5 (4.3) | 15 (51.7) | < 0.001 |
|  | Facility readiness and stock tracking | | 2 (1.5) | 2 (1.7) | 0 (0.0) | 1.000 |
|  | Healthcare worker training and monitoring | | 12 (9.2) | 17 (14.5) | 2 (6.9) | 0.46 |
|  | Entry and follow-up of laboratory tests | | 27 (20.8) | 4 (3.4) | 13 (44.8) | < 0.001 |
|  | Event-based surveillance | | 77 (59.2) | 31 (26.5) | 14 (48.3) | 0.004 |
|  | Reporting | | 69 (53.1) | 80 (68.4) | 15 (51.7) | 0.44 |
|  | Informing patients | | 13 (10.0) | 5 (4.3) | 4 (13.8) | 0.12 |
|  | Clinical management of cases | | 19 (14.6) | 17 (14.5) | 7 (24.1) | 0.50 |
